# Mechanistic Insights into Vorinostat as a Repositioned Modulator of TACE-Mediated TNF-α Signaling via MAPK and NFκB Pathways

**DOI:** 10.3390/cimb47090720

**Published:** 2025-09-04

**Authors:** Jinyoung Park, Muhammad Yasir, Jongseon Choe, Jin-Hee Han, Eun-Taek Han, Won Sun Park, Wanjoo Chun

**Affiliations:** 1 Department of Pharmacology, Kangwon National University School of Medicine, Chuncheon 24341, Republic of Korea; jinyoung0326@kangwon.ac.kr (J.P.); yasir.khokhar1999@gmail.com (M.Y.); 2 Department of Microbiology and Immunology, Kangwon National University School of Medicine, Chuncheon 24341, Republic of Korea; jchoe@kangwon.ac.kr; 3 Department of Medical Environmental Biology and Tropical Medicine, Kangwon National University School of Medicine, Chuncheon 24341, Republic of Korea; han.han@kangwon.ac.kr (J.-H.H.); ethan@kangwon.ac.kr (E.-T.H.); 4 Department of Physiology, Kangwon National University School of Medicine, Chuncheon 24341, Republic of Korea; parkws@kangwon.ac.kr

**Keywords:** vorinostat, TACE (ADAM17), TNF-α, MAPK/NFκB, drug repositioning

## Abstract

Vorinostat, an FDA-approved histone deacetylase inhibitor, was evaluated for its potential anti-inflammatory activity through modulation of TACE (ADAM17)-mediated TNF-α signaling. The study was conducted using LPS-stimulated RAW264.7 macrophages. TACE enzymatic activity was assessed by a fluorogenic assay, TNF-α release was measured by ELISA, and phosphorylation of MAPKs and NFκB signaling proteins was examined by a western blot. Molecular docking was performed using GNINA to evaluate binding affinity to ERK. Vorinostat was found to modestly inhibit TACE enzymatic activity in vitro, while significantly suppressing TNF-α secretion in cells, comparable to the selective TACE inhibitor BMS-561392. A concentration-dependent reduction in phosphorylated IκB and NFκB was observed, along with selective inhibition of ERK phosphorylation. Docking studies indicated a stable, albeit weaker, binding of vorinostat to ERK compared to reference ERK inhibitors. These findings suggest that vorinostat suppresses TNF-α production primarily through indirect mechanisms involving ERK and NF-κB signaling pathways, rather than by direct TACE inhibition. The repositioning of vorinostat as a modulator of inflammatory signaling is supported, offering potential therapeutic value in inflammatory disorders.

## 1. Introduction

In recent years, drug repositioning has gained significant momentum as a strategic alternative to conventional de novo drug development [1,2,3]. Also known as drug repurposing, this approach involves identifying new therapeutic uses for existing drugs that are already approved for other indications [4,5]. The advantages of drug repositioning are substantial: it drastically reduces the time and cost required for drug discovery [5,6], since pharmacokinetic profiles, toxicity data, and safety margins are already established [7]. Moreover, the regulatory pathways for repositioned drugs are often accelerated, and the clinical trial failure rates are significantly lower compared to novel compounds [8]. This strategy is particularly attractive for addressing diseases with unmet medical needs or for expanding treatment options for complex pathologies such as cancer, neurodegeneration, and chronic inflammation.

One such complex therapeutic target is the tumor necrosis factor-α converting enzyme (TACE), also known as ADAM17 (A Disintegrin and Metalloproteinase 17) [9]. TACE is a membrane-bound metalloprotease that mediates the ectodomain shedding of various membrane proteins, including pro-TNF-α, IL-6 receptors, and EGF-family ligands [10,11]. Through this shedding process, TACE regulates the bioavailability of several key cytokines and growth factors that influence immune responses, tissue remodeling, and inflammation [11,12,13]. Dysregulated TACE activity has been implicated in a wide range of pathologies, such as rheumatoid arthritis, psoriasis, inflammatory bowel disease, sepsis, and cancer [10,14,15,16]. Consequently, selective inhibition of TACE has been proposed as a promising therapeutic strategy, and several small molecule inhibitors have been developed [17,18]. However, many of these compounds have failed to progress clinically due to issues such as off-target effects, poor selectivity, and toxicity [19,20,21].

In our previous work, Discovery of novel TACE inhibitors using graph convolutional network, molecular docking, molecular dynamics simulation, and biological evaluation we sought to address these challenges through a computational drug discovery pipeline [22]. We utilized graph convolutional networks (GCN) to screen compound libraries, followed by performing structure-based docking and molecular dynamics simulations to identify potential TACE inhibitors. Among the compounds identified, vorinostat (suberoylanilide hydroxamic acid, SAHA) emerged as a promising hit. Vorinostat is a well-characterized histone deacetylase (HDAC) inhibitor approved by the FDA for the treatment of cutaneous T-cell lymphoma (CTCL) [23,24,25]. Interestingly, in vitro evaluation revealed that vorinostat could reduce TNF-α expression in LPS-stimulated RAW264.7 macrophages, suggesting that it may possess previously unrecognized anti-inflammatory activity potentially linked to TACE regulation [22].

Vorinostat has been extensively studied for its epigenetic effects in cancer, where it modulates gene expression by inhibiting the deacetylation of histone and non-histone proteins [26,27]. However, accumulating evidence indicates that HDAC inhibitors also exert anti-inflammatory effects by modulating signaling pathways such as NFκB [28], MAPK [29,30], and STATs, all of which are involved in cytokine gene regulation [31,32,33,34]. The observation that vorinostat may influence TACE-mediated TNF-α release highlights the need to clarify whether its mechanism involves a direct inhibition of TACE or an indirect effect through upstream signaling modulation or transcriptional regulation. Preliminary enzymatic assays suggest that vorinostat does not significantly inhibit TACE catalytic activity in vitro, thereby supporting the possibility that it functions through indirect regulatory mechanisms, potentially by suppressing TNF-α gene expression or altering TACE activation via MAPK signaling.

In this study, we aim to explore the mechanistic basis of vorinostat’s anti-inflammatory action in RAW264.7 cells, with particular focus on its effects on TACE activity, MAPK pathway signaling, NFκB phosphorylation, and TNF-α production. By dissecting these molecular interactions, we seek to clarify whether vorinostat acts as a direct TACE inhibitor or an indirect modulator of TNF-α shedding. Ultimately, this study not only provides insight into the repositioning potential of vorinostat as a therapeutic agent for inflammatory diseases but also offers a deeper mechanistic understanding of how clinically approved drugs can modulate key inflammatory pathways, thereby expanding their therapeutic utility.

## 2. Materials and Methods

### 2.1. Reagents and Cell Culture

Bacterial lipopolysaccharide (LPS) from Escherichia coli serotype 055:B5 was purchased from Sigma-Aldrich (St. Louis, MO, USA). BMS-561392 formate was purchased from MedChemExpress (Monmouth Junction, NJ, USA). Vorinostat was obtained from Sigma-Aldrich (St. Louis, MO, USA). Raw 264.7 macrophage cells were supplied by the Korea cell line bank (KCLB, cat #40071). The cells were cultured in Dulbecco’s modified Eagle’s medium (DMEM; BYLABS, Luscience Corporation, Hanam-si, Republic of Korea) containing 10% heat-inactivated fetal bovine serum and 100 U/mL penicillin-streptomycin (Gibco), under standard conditions of 37 °C in a humidified atmosphere containing 5% CO_2_. The cells were pre-treated with the indicated concentrations of the reagents for 1 h, followed by stimulation with 1 μg/mL of LPS for 4 h.

### 2.2. ELISA Assay for TNF-α

Raw 264.7 macrophage cells were pretreated with BMS-561392 and vorinostat (10 μM) for 1 h and then stimulated with 1 μg/mL of LPS for 4 h. Tumor necrosis factor-α (TNF-α) levels in culture media were quantified using enzyme-linked immunosorbent (ELISA) kits (R&D systems, Minneapolis, MN 55413, USA) according to the manufacturer’s instructions.

### 2.3. ELISA Assay for TACE Activity

TACE activity was measured using the ADAM17 Fluorogenic Assay Kit (BPS Bioscience, Cat. #78000, San Diego, CA, USA) following the manufacturer’s protocol. Briefly, recombinant ADAM17 enzyme was incubated with a fluorogenic peptide substrate in black 96-well plates in a final volume of 50 μL/well. Test compounds, including vorinostat or a reference TACE inhibitor (BMS-561392), were prepared and diluted to the appropriate concentrations using the assay buffer provided in the kit. The reaction mixture contained 20 μL of ADAM17 enzyme solution, 5 μL of test inhibitor, and 25 μL of ADAM Fluorogenic Substrate. The plate was incubated at room temperature for 1 h, 2 h, and 2.5 h. After incubation, fluorescence intensity was measured using a microplate reader at excitation of 485 nm and emission of 530 nm. Enzyme activity was calculated based on the relative fluorescence units (RFU) compared to the vehicle control group. All reactions were performed in triplicate, and data were presented as mean ± SD.

### 2.4. Western Blot Analysis

Raw 264.7 macrophage cells were treated with either BMS-561392 or vorinostat (1, 10, 100 μM) for 1 h, followed by stimulation with LPS (1 μg/mL). After treatment cells were rinsed with PBS and lysed using PRO-PREP lysis buffer (iNtRON Biotechnology, Seongnam-si, Gyeonggi-do, Repunlic of Korea), equal amounts of total protein were subjected to 10% SDS-polyacrylamide gel electrophoresis and subsequently transferred to Hypond PVDF membrane (Merck KGaA, Darmstadt, Germany). Membranes were blocked for 1 h at room temperature with 5% skim milk in tris-buffered saline with tween20 (TBST). Primary antibodies against p-IκB, IκB, p-NFκB, NFκB, p-ERK, ERK, p-JNK, JNK, p-p38, and p38 (1:1000; Cell signaling Technology, Danvers, MA, USA) as well as β-actin (1:1000; Santa Cruz Biotechnology Inc., Dallas, TX, USA) were diluted in blocking solution and applied overnight. After washing with TBST, membranes were incubated with HRP-conjugated secondary antibodies. Protein bands were visualized using enhanced chemiluminescence (ECL; Abfrontier, San Jose, CA, USA), and images were acquired with a FUSION SOLO S imaging system (Vilber, ZAC de Lamirault, Collegien, Marne-la-vallee, France).

### 2.5. Molecular Docking

The 3D structure of ERK was obtained from the Protein Data Bank (PDB) database (https://www.rcsb.org/, accessed on 26 May 2025). Docking simulations were performed using GNINA 1.1, a deep learning-based molecular docking platform that employs convolutional neural networks to enhance scoring accuracy for protein–ligand interactions. GNINA has demonstrated superior performance in virtual screening tasks compared to conventional scoring functions such as AutoDock Vina, providing improved early enrichment and pose prediction accuracy [35,36]. GNINA scores, expressed in kcal/mol, represent the predicted binding affinities between ligands and the target protein, with more negative values indicating stronger binding potential. To complement the docking results, a detailed analysis of key protein–ligand interactions was carried out for selected compounds (SCH772984, tizaterkib, and vorinostat) using Discovery Studio (version 22) and UCSF Chimera (version 1.16).

### 2.6. Statistical Analysis

All data presented in the figures are expressed as the mean ± standard deviation (SD) derived from a minimum of three independent experiments. Statistical evaluation was performed using two-tailed Student’s *t*-test with *p*-values less than 0.05 regarded as statistically significant. Significance levels are indicated in the figures by single symbols (*^,#^) for *p* < 0.05 and double symbols (**^, ##^) for *p* < 0.01.

## 3. Results

### 3.1. Vorinostat Modestly Inhibited TACE (ADAM17) Enzymatic Activity

**Figure 1:**
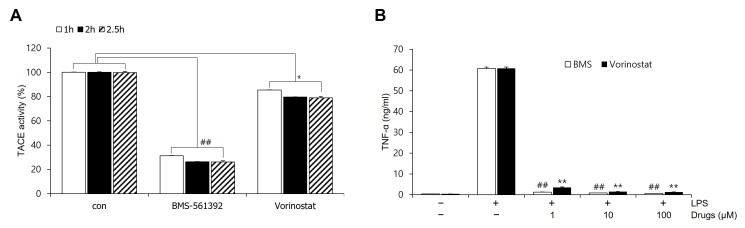
Effect of vorinostat on TACE (ADAM17) activity and TNF-α expression. (**A**) TACE activity was evaluated using a fluorogenic ADAM17 assay in the presence of 10 nM BMS-561392 or 10 nM vorinostat. The assay mixture was incubated with each inhibitor for 1, 2, or 2.5 h. * *p* < 0.05 vs. control in vorinostat treated set; ## *p* < 0.001 vs. control in BMS-561392 treated set. (**B**) TNF-α expression was measured by ELISA following LPS stimulation (1 μg/mL, 4 h) in RAW264.7 cells pretreated with BMS-561392 or vorinostat at concentrations of 1, 10, or 100 μM. ** *p* < 0.001 vs. LPS alone in vorinostat treated set; ## *p* < 0.001 vs. LPS alone in BMS-561392 treated set. Data are presented as mean ± SD (n = 3).

To evaluate the inhibitory effects of vorinostat and the reference inhibitor BMS-561392 on TACE (ADAM17) enzymatic activity, we performed an in vitro fluorogenic assay using the ADAM17 Fluorogenic Assay Kit. Recombinant human ADAM17 enzyme and fluorogenic substrate were incubated with BMS-561392 or vorinostat in 96-well plates under standard assay conditions. The enzymatic activity was quantified based on fluorescence intensity generated by substrate cleavage. BMS-561392 significantly inhibited ADAM17 activity, reducing fluorescence by approximately 80% compared to the vehicle control. In contrast, vorinostat resulted in only a modest reduction (~20%), suggesting weak or indirect inhibition of ADAM17 catalytic activity (Figure 1A). To assess whether enzyme inhibition varied with incubation time, the assay was conducted at 1 h, 2 h, and 2.5 h. BMS-561392 consistently and significantly suppressed TACE enzymatic activity across all time points. Vorinostat also reduced TACE activity relative to the untreated control at each time point, and the reduction reached statistical significance, although the extent of inhibition was consistently lower than that observed with BMS-561392. No significant time-dependent differences in fluorescence signals were detected for either compound.

### 3.2. Vorinostat Inhibited LPS-Induced TNF-α Release

We evaluated soluble TNF-α levels as a functional indicator of TACE activity. Stimulation with LPS markedly elevated TNF-α release in RAW264.7 cells, with levels reaching approximately 60 ng/mL, in contrast to the low basal levels observed in the untreated control. Notably, pre-treatment with either BMS-561392 or vorinostat significantly attenuated LPS-induced TNF-α production. In both treatment groups, TNF-α levels were reduced to near-control levels, indicating effective suppression of inflammatory cytokine release (Figure 1B). This inhibitory effect was observed consistently across all tested concentrations (1, 10, and 100 μM), confirming that even at the lowest concentration tested, both compounds were sufficient to suppress TNF-α release effectively.

### 3.3. Vorinostat Attenuated LPS-Induced IκB Phosphorylation

**Figure 2:**
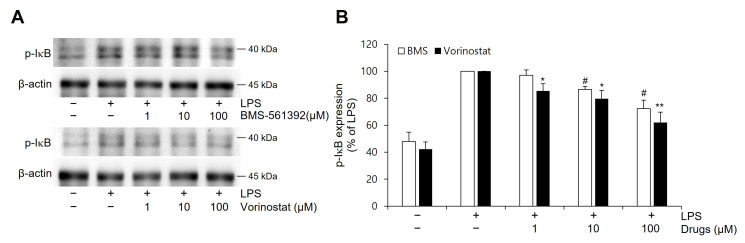
Effect of vorinostat on LPS-induced phosphorylation of IκB expression in RAW264.7 cells. (**A**) Representative immunoblots. (**B**) Quantitative analyses of immunoblots of p-IκB. Cells were pretreated with vorinostat (1, 10, and 100 μM) or BMS-561392 (1, 10, and 100 μM) for 1 h and then stimulated with LPS for 4 h. Vorinostat exhibited a significant attenuation of LPS-induced p-IκB expression in a concentration-dependent manner. The data presented are the means ± SD of three independent experiments. * *p* < 0.05, ** *p* < 0.001 vs. LPS alone in vorinostat treated set; # *p* < 0.05, vs. LPS alone in BMS-561392 treated set.

To investigate the effects of BMS-561392 and vorinostat on NFκB pathway activation, the expression level of phosphorylated IκB was measured in LPS-induced RAW264.7 cells by western blot analysis (Figure 2). LPS stimulation markedly increased p-IκB expression compared to the control group, indicating robust activation of the NFκB signaling cascade. Pre-treatment with either BMS-561392 or vorinostat resulted in a dose-dependent reduction in p-IκB levels, suggesting inhibition of LPS-induced NFκB activation. Notably, vorinostat exhibited a more pronounced suppressive effect on p-IκB expression than BMS-561392 at equivalent concentrations.

### 3.4. Vorinostat Suppressed LPS-Induced NFκB Phosphorylation

**Figure 3:**
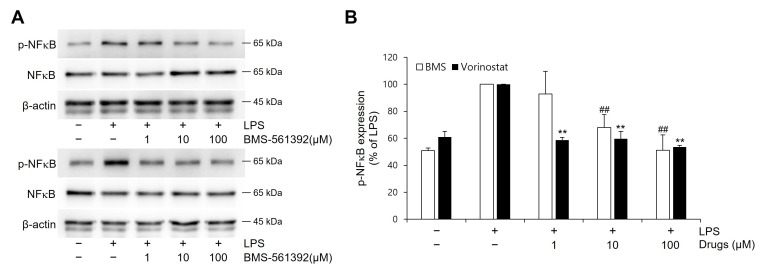
Effect of vorinostat on LPS-induced phosphorylation of NFκB in RAW264.7 cells. Cells were pretreated with vorinostat or BMS-561392 (1, 10, and 100 μM) for 1 h and then stimulated with LPS for 1 h. (**A**) representative immunoblots of p-NFκB and NFκB. (**B**) quantitative analyses of immunoblots of p-NFκB. The data presented are the means ± SD of three independent experiments. ** *p* < 0.001 vs. LPS alone in vorinostat treated set; ## *p* < 0.001 vs. LPS alone in BMS-561392 treated set.

The levels of phosphorylated NFκB were examined in LPS-stimulated RAW 264.7 cells (Figure 3). In comparison to the unstimulated control, LPS treatment increased p-NFκB. Both BMS-561392 and vorinostat effectively attenuated this LPS-induced phosphorylation, particularly suppressing p-NFκB expression even at lower concentrations.

### 3.5. Vorinostat Significantly Suppressed LPS-Induced MAPKs

**Figure 4:**
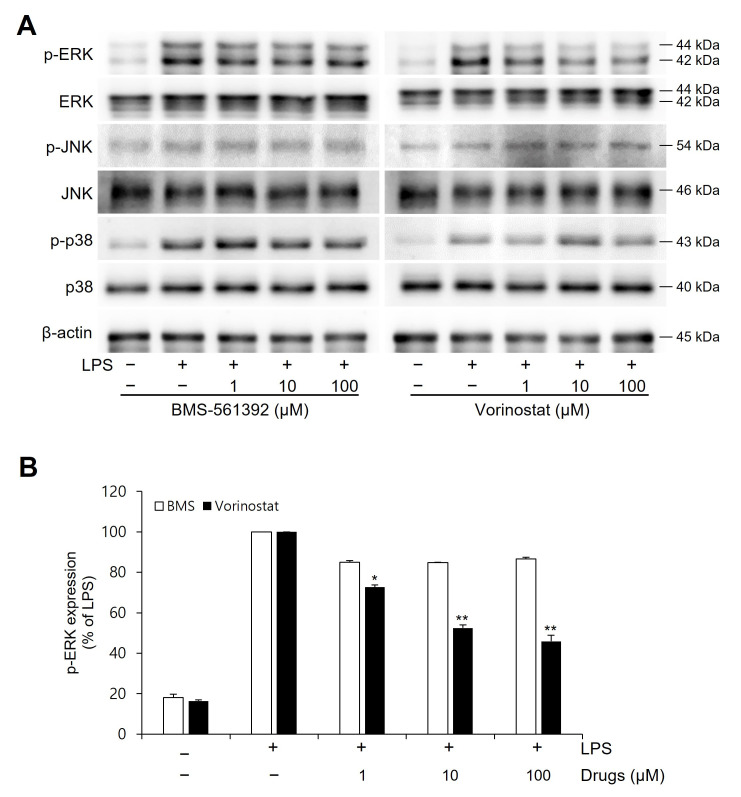
Effect of vorinostat on LPS-induced phosphorylation of MAPKs in RAW264.7 cells. Cells were pretreated with vorinostat or BMS-561392 (1, 10, and 100 μM) for 1 h and then stimulated with LPS for 1 h. (**A**) Representative immunoblots of MAPKs. (**B**) Quantitative analyses of immunoblots of p-ERK. The data presented are the means ± SD of three independent experiments. * *p* < 0.05, ** *p* < 0.001 vs. LPS alone in vorinostat-treated set.

To further explore the effects of vorinostat and BMS-561392 on upstream inflammatory signaling pathways, we examined the activation of key MAPK pathways by assessing the phosphorylation levels of ERK, JNK, and p38 in LPS-stimulated RAW264.7 cells (Figure 4). LPS treatment strongly induced phosphorylation of all three MAPKs, confirming activation of the signaling cascade. Vorinostat markedly suppressed p-ERK levels by approximately 55%, although BMS-561392 exhibited only a modest reduction of around 20%. However, no significant changes were observed in p-JNK or p-p38 expression following treatment with either compound.

### 3.6. Molecular Docking

To evaluate the binding affinity of vorinostat toward ERK, molecular docking simulations were performed using two 3D ERK crystal structures, 4QTA and 6SLG, via the GNINA docking platform. A panel of clinically relevant ERK inhibitors-SCH772984, ulixertinib (BVD-523), tizaterkib (AZD0364), and temuterkib (LY3214996) was included as reference ligands for comparative analysis. In the 4QTA docking, SCH772984 exhibited the strongest binding affinity (−12.16 kcal/mol), followed by tizaterkib, ulixertinib, and temuterkib. Vorinostat exhibited a comparatively weaker binding energy of −7.71 kcal/mol. Similarly, docking to 6SLG revealed consistent trends: SCH772984 again ranked highest (−9.17 kcal/mol), with other ERK inhibitors displaying binding energies in the range of −8.99 to −9.72 kcal/mol. Vorinostat showed a relatively weak affinity at −6.44 kcal/mol. This result showed that, despite demonstrating lower binding affinities relative to reference ERK inhibitors, vorinostat’s docking scores remained within the range generally considered compatible with meaningful ligand–target engagement in silico.

### 3.7. Molecular Docking Interactions

**Figure 5:**
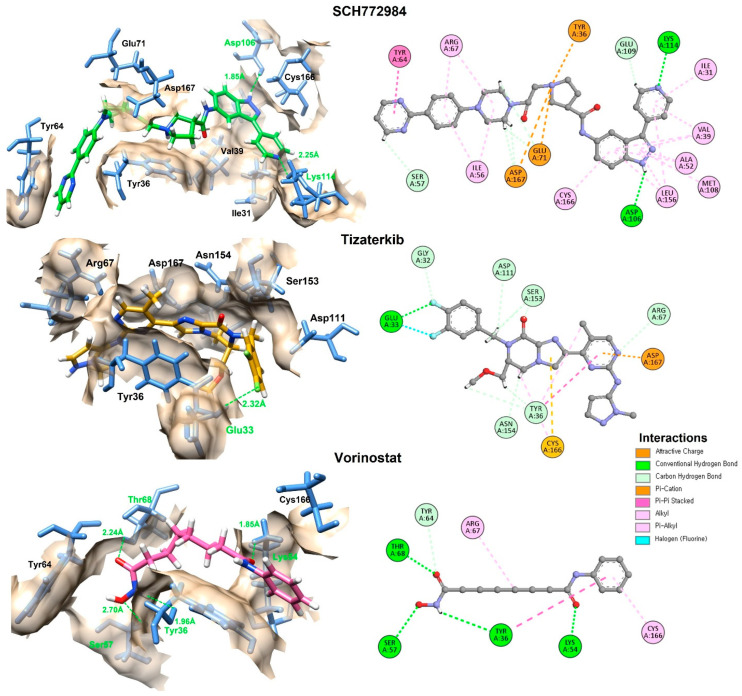
The 2D and 3D interactions of the top two docked compounds against the active region of the ERK in comparison with vorinostat. The hydrogen bonds are colored green, while the other amino acids interacting hydrophobically and electrostatically are also displayed.

A detailed molecular docking interaction analysis was carried out with the GNINA docked complexes using Discovery Studio and UCSF Chimera visualization tools. The interactions were evaluated based on hydrogen bonds, hydrophobic interactions, and electrostatic interactions with the binding pocket residues of the target protein (Figure 5). Table 1 summarizes the hydrogen bond-forming amino acid residues and their respective binding distances.

The interaction analysis reveals that SCH772984, which exhibited the most favorable molecular docking energy (Table 2), formed hydrogen bonds with Asp106 and Lys114 at close distances of 1.85 Å and 2.25 Å, respectively. These interactions were complemented by favorable electrostatic and hydrophobic contacts, which allowed the compound to anchor firmly within the catalytic cleft and achieve a stable conformation. Tizaterkib, the second-best performer in the docking analysis, formed a hydrogen bond with Glu33 (2.32 Å), further stabilized by multiple electrostatic interactions that anchored it to different binding pocket residues, contributing to its strong affinity. Vorinostat, although ranked lower in docking energy, surprisingly showed the highest number of hydrogen bond interactions (four residues: Thr68, Lys54, Tyr36, and Ser57) with distances ranging from 1.85 Å to as 2.70 Å. However, despite its extensive hydrogen bonding network, the absence of multiple hydrophobic and electrostatic anchoring interactions likely reduces its overall stability in the binding pocket. This suggests that vorinostat, while capable of forming multiple hydrogen bonds, may adopt a binding conformation that does not fit as snugly into the hydrophobic core compared to SCH772984 and tizaterkib, resulting in a less favorable docking score.

## 4. Discussion

In our previous study, we identified vorinostat as a potential TACE (ADAM17) inhibitor through a drug repositioning approach using computational and biological screening strategies [22]. However, the precise mechanism by which vorinostat exerts its anti-inflammatory effects remained unclear. Specifically, it was uncertain whether vorinostat directly inhibits TACE enzymatic activity or instead modulates upstream signaling pathways that affect the production and release of TNF-α. To address this question, the current study was conducted to evaluate vorinostat’s mode of action at multiple regulatory levels in the TNF-α signaling cascade.

The initial assessment of TACE enzymatic activity using an in vitro fluorogenic assay revealed that vorinostat did not inhibit ADAM17 activity to the same extent as the reference inhibitor BMS-561392. While BMS-561392 reduced enzymatic activity by approximately 80%, vorinostat elicited only a modest inhibition (~20%), suggesting that vorinostat does not function as a strong direct inhibitor of TACE under cell-free conditions. These results reinforce the classification of BMS-561392 as a direct enzymatic inhibitor and imply that vorinostat may exert its regulatory effects through alternative, possibly indirect mechanisms. Given TACE’s established role in cleaving membrane-bound pro-TNF-α to release its soluble, biologically active form, we evaluated TNF-α secretion as a functional readout of TACE activity in LPS-stimulated RAW264.7 macrophages. Both BMS-561392 and vorinostat significantly suppressed TNF-α release. Notably, vorinostat achieved comparable or even superior suppression at lower concentrations, despite its limited direct enzymatic inhibition in vitro. This discrepancy suggests that vorinostat may effectively reduce TNF-α secretion in a cellular context by modulating upstream regulatory mechanisms, including transcriptional and post-translational control of TNF-α production.

Western blot analysis further elucidated these regulatory effects. Vorinostat markedly inhibited LPS-induced phosphorylation of IκBα and NFκB p65, two critical events in the activation of the NFκB pathway. Across tested concentrations, vorinostat demonstrated a reproducible trend toward stronger suppression than BMS-561392. As NFκB is a master regulator of inflammatory gene transcription—including TNF-α—these findings suggest that vorinostat attenuates inflammation by interfering with both TNF-α transcription and its release via the TACE pathway. While our study was not primarily designed to establish direct statistical comparisons of potency between vorinostat and BMS-561392, the biological consistency across experiments highlights vorinostat’s potential as a repositioned anti-inflammatory agent. Furthermore, we observed that vorinostat selectively suppressed phosphorylation of ERK—an essential kinase in the MAPK pathway—by approximately 55%. In contrast, BMS-561392 produced only mild ERK inhibition (~20%), and neither compound significantly altered p-JNK nor p-p38 levels. Since ERK activation contributes to both NFκB signaling and TNF-α gene expression, this ERK-selective suppression by vorinostat may explain its enhanced anti-inflammatory profile relative to BMS-561392. Notably, recent studies have highlighted the importance of MAPK pathway regulation in modulating inflammatory and oncogenic responses—for example, parecoxib’s enhancement of resveratrol activity through MAPK modulation [37]. This broader context further supports our interpretation that selective ERK suppression represents a critical component of vorinostat’s anti-inflammatory mechanism.

In addition to the ERK and NFκB pathways characterized here, vorinostat’s well-established role as an HDAC inhibitor suggests that epigenetic regulation may also contribute to the observed suppression of TNF-α production. HDAC inhibition increases histone acetylation, thereby relaxing chromatin structure and attenuating transcription of pro-inflammatory genes, including TNF-α. Previous studies have shown that HDAC inhibitors broadly reduce cytokine production in macrophages by this mechanism [31,33]. Thus, vorinostat’s anti-inflammatory profile in our model may reflect both direct modulation of signaling cascades and indirect transcriptional control mediated by canonical HDAC-dependent pathways.

TACE (ADAM17) activity is influenced not only by its catalytic site but also by expression and membrane localization, which are regulated by MAPKs and post-translational modifications. Although not directly assessed here, vorinostat’s suppression of ERK and NFκB suggests that its effects extend beyond enzymatic inhibition to include transcriptional regulation of TNF-α. This aligns with the canonical activity of HDAC inhibitors, which reduce cytokine expression via chromatin remodeling.

A limitation of this study is that vorinostat was tested at micromolar concentrations (1–100 μM), which exceed clinically achievable plasma levels. Although higher doses are often needed in vitro due to uptake, binding, and stability differences, this raises uncertainty for clinical translation. Moreover, our acute macrophage model does not capture the complexity of in vivo immune regulation. Future studies in primary human cells, in vivo models, and PK/PD analyses will be required to confirm efficacy at clinically relevant exposures.

**Figure 6:**
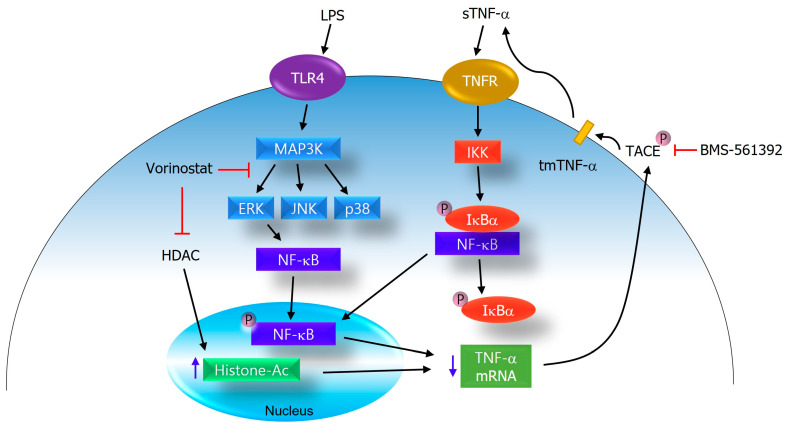
Proposed mechanism of action for vorinostat in the regulation of TNF-α production. Vorinostat suppresses inflammatory signaling by attenuating ERK and NFκB activation and indirectly modulating TACE activity. In addition, as a canonical HDAC inhibitor, vorinostat increases histone acetylation, which contributes to a reduced transcription of pro-inflammatory genes including TNF-α. Together, these canonical and non-canonical mechanisms converge to disrupt TNF-α production and inflammatory amplification in LPS-stimulated RAW264.7 cells.

In summary, although vorinostat does not act as a potent direct inhibitor of TACE enzymatic activity in vitro, it demonstrates significant anti-inflammatory efficacy through a multifactorial mechanism involving inhibition of TNF-α gene expression, suppression of NFκB and ERK signaling, and potential indirect modulation of TACE activity (Figure 6). These findings highlight vorinostat’s therapeutic potential as a repositioned agent for inflammatory diseases and underscore the importance of evaluating both direct and indirect pathways when characterizing the mechanism of action for repositioned drugs. It should be acknowledged that vorinostat is basically an HDAC inhibitor, and whether its anti-inflammatory effects arise from canonical HDAC-dependent mechanisms (e.g., histone acetylation and transcriptional regulation) or off-target actions remains unresolved. Given that HDAC inhibition is well established in the literature, our study focused on novel TACE- and signaling-related mechanisms. Nonetheless, the lack of direct HDAC activity assays or comparisons with other HDAC inhibitors is a limitation. Future work using HDAC activity measurements and selective controls will be essential to clarify the relative contributions of HDAC-dependent and independent pathways to vorinostat’s effects.

## Figures and Tables

**Table 1:** Molecular docking energies of vorinostat and reference ERK inhibitors.

		Binding Energy (kcal/mol)	
	Target protein (PDB ID)	ERK (4QTA)	ERK (6SLG)
Inhibitors			
SCH772984		−12.16	−9.17
Ulixertinib (BVD-523)		−9.91	−9.04
Tizaterkib (AZD0364)		−10.75	−9.72
Temuterkib (LY3214996)		−9.83	−8.99
vorinostat		−7.71	−6.44

**Table 2:** In the molecular docking interactions of the tested compounds, hydrogen bonds and hydrogen bonding distances are manifested.

Compounds	Interacting Residues	Binding Distances
SCH772984	Asp106	1.85 Å
	Lys114	2.25 Å
Tizaterkib	Glu33	2.32 Å
Vorinostat	Thr68	2.24 Å
	Lys54	1.85 Å
	Tyr36	1.96 Å
	Ser57	2.70 Å

## Data Availability

Raw 264.7 macrophage cells were supplied by the Korea cell line bank (KCLB, cat #40071). The data that support the findings of this study are available from the corresponding author upon reasonable request.

## References

[1] Parvathaneni V., Kulkarni N.S., Muth A., Gupta V. (2019). Drug repurposing: A promising tool to accelerate the drug discovery process. Drug Discov. Today.

[2] Low Z.Y., Farouk I.A., Lal S.K. (2020). Drug Repositioning: New Approaches and Future Prospects for Life-Debilitating Diseases and the COVID-19 Pandemic Outbreak. Viruses.

[3] Kumbhar P., Kole K., Yadav T., Bhavar A., Waghmare P., Bhokare R., Manjappa A., Jha N.K., Chellappan D.K., Shinde S. (2022). Drug repurposing: An emerging strategy in alleviating skin cancer. Eur. J. Pharmacol..

[4] Azad I., Khan T., Ahmad N., Khan A.R., Akhter Y. (2023). Updates on drug designing approach through computational strategies: A review. Future Sci. OA.

[5] Shah F.A., Qadir H., Khan J.Z., Faheem M. (2025). A review: From old drugs to new solutions: The role of repositioning in alzheimer’s disease treatment. Neuroscience.

[6] Nosengo N. (2016). Can you teach old drugs new tricks?. Nature.

[7] Pushpakom S., Iorio F., Eyers P.A., Escott K.J., Hopper S., Wells A., Doig A., Guilliams T., Latimer J., McNamee C. (2019). Drug repurposing: Progress, challenges and recommendations. Nat. Rev. Drug Discov..

[8] Ashburn T.T., Thor K.B. (2004). Drug repositioning: Identifying and developing new uses for existing drugs. Nat. Rev. Drug Discov..

[9] Black R.A., White J.M. (1998). ADAMs: Focus on the protease domain. Curr. Opin. Cell Biol..

[10] Scheller J., Chalaris A., Garbers C., Rose-John S. (2011). ADAM17: A molecular switch to control inflammation and tissue regeneration. Trends Immunol..

[11] Zunke F., Rose-John S. (2017). The shedding protease ADAM17: Physiology and pathophysiology. Biochim. Biophys. Acta Mol. Cell Res..

[12] Sun Q., Hampel H., Blennow K., Lista S., Levey A., Tang B., Li R., Shen Y. (2014). Increased plasma TACE activity in subjects with mild cognitive impairment and patients with Alzheimer’s disease. J. Alzheimers Dis..

[13] Bahia M.S., Silakari O. (2010). Tumor necrosis factor alpha converting enzyme: An encouraging target for various inflammatory disorders. Chem. Biol. Drug Des..

[14] Moss M.L., Lambert M.H. (2002). Shedding of membrane proteins by ADAM family proteases. Essays Biochem..

[15] Inam Illahi M., Amjad S., Alam S.M., Ahmed S.T., Fatima M., Shahid M.A. (2021). Serum Tumor Necrosis Factor-Alpha as a Competent Biomarker for Evaluation of Disease Activity in Early Rheumatoid Arthritis. Cureus.

[16] Farrugia M., Baron B. (2016). The role of TNF-alpha in rheumatoid arthritis: A focus on regulatory T cells. J. Clin. Transl. Res..

[17] Li N.G., Shi Z.H., Tang Y.P., Wei L., Lian Y., Duan J.A. (2012). Discovery of selective small molecular TACE inhibitors for the treatment of rheumatoid arthritis. Curr. Med. Chem..

[18] Murumkar P.R., DasGupta S., Chandani S.R., Giridhar R., Yadav M.R. (2010). Novel TACE inhibitors in drug discovery: A review of patented compounds. Expert. Opin. Ther. Pat..

[19] Dusterhoft S., Lokau J., Garbers C. (2019). The metalloprotease ADAM17 in inflammation and cancer. Pathol. Res. Pr..

[20] Moss M.L., Minond D. (2017). Recent Advances in ADAM17 Research: A Promising Target for Cancer and Inflammation. Mediat. Inflamm..

[21] Murumkar P.R., Giridhar R., Yadav M.R. (2013). Novel methods and strategies in the discovery of TACE inhibitors. Expert. Opin. Drug Discov..

[22] Yasir M., Park J., Han E.T., Han J.H., Park W.S., Hassan M., Kloczkowski A., Chun W. (2024). Discovery of novel TACE inhibitors using graph convolutional network, molecular docking, molecular dynamics simulation, and Biological evaluation. PLoS ONE.

[23] Duvic M., Vu J. (2007). Vorinostat: A new oral histone deacetylase inhibitor approved for cutaneous T-cell lymphoma. Expert. Opin. Investig. Drugs.

[24] Pili R., Liu G., Chintala S., Verheul H., Rehman S., Attwood K., Lodge M.A., Wahl R., Martin J.I., Miles K.M. (2017). Combination of the histone deacetylase inhibitor vorinostat with bevacizumab in patients with clear-cell renal cell carcinoma: A multicentre, single-arm phase I/II clinical trial. Br. J. Cancer.

[25] Marks P.A. (2010). HDAC inhibitors: Much to learn about effective therapy. Oncology.

[26] Lee J., R S.H. (2013). Cancer Epigenetics: Mechanisms and Crosstalk of a HDAC Inhibitor, Vorinostat. Chemotherapy.

[27] Richon V.M. (2010). Targeting histone deacetylases: Development of vorinostat for the treatment of cancer. Epigenomics.

[28] Bolden J.E., Peart M.J., Johnstone R.W. (2006). Anticancer activities of histone deacetylase inhibitors. Nat. Rev. Drug Discov..

[29] Scott A.J., O’Dea K.P., O’Callaghan D., Williams L., Dokpesi J.O., Tatton L., Handy J.M., Hogg P.J., Takata M. (2011). Reactive oxygen species and p38 mitogen-activated protein kinase mediate tumor necrosis factor alpha-converting enzyme (TACE/ADAM-17) activation in primary human monocytes. J. Biol. Chem..

[30] Xu P., Derynck R. (2010). Direct activation of TACE-mediated ectodomain shedding by p38 MAP kinase regulates EGF receptor-dependent cell proliferation. Mol. Cell.

[31] Leoni F., Fossati G., Lewis E.C., Lee J.K., Porro G., Pagani P., Modena D., Moras M.L., Pozzi P., Reznikov L.L. (2005). The histone deacetylase inhibitor ITF2357 reduces production of pro-inflammatory cytokines in vitro and systemic inflammation in vivo. Mol. Med..

[32] Fang S., Meng X., Zhang Z., Wang Y., Liu Y., You C., Yan H. (2016). Vorinostat Modulates the Imbalance of T Cell Subsets, Suppresses Macrophage Activity, and Ameliorates Experimental Autoimmune Uveoretinitis. Neuromolecular Med..

[33] Adcock I.M. (2007). HDAC inhibitors as anti-inflammatory agents. Br. J. Pharmacol..

[34] Halili M.A., Andrews M.R., Sweet M.J., Fairlie D.P. (2009). Histone deacetylase inhibitors in inflammatory disease. Curr. Top. Med. Chem..

[35] McNutt A.T., Francoeur P., Aggarwal R., Masuda T., Meli R., Ragoza M., Sunseri J., Koes D.R. (2021). GNINA 1.0: Molecular docking with deep learning. J. Cheminform.

[36] Sunseri J., Koes D.R. (2021). Virtual Screening with Gnina 1.0. Molecules.

[37] Chang W.L., Yang K.C., Peng J.Y., Hong C.L., Li P.C., Chye S.M., Lu F.J., Shih C.W., Chen C.H. (2024). Parecoxib Enhances Resveratrol against Human Colorectal Cancer Cells through Akt and TXNDC5 Inhibition and MAPK Regulation. Nutrients.

